# Recent Interventions for Acute Suicidality Delivered in the Emergency Department: A Scoping Review

**DOI:** 10.5811/westjem.53023

**Published:** 2025-10-03

**Authors:** Alex P. Hood, Lauren M. Tibbits, Juan I. Laporta, Jennifer Carrilo, Lacee R. Adams, Stacey Young-McCaughan, Alan L. Peterson, Robert A. DeLorenzo

**Affiliations:** * University of Texas Health Science Center at San Antonio, Department of Emergency Medicine, San Antonio, Texas; † Baylor University, Department of Psychology and Neuroscience, Waco, Texas; ‡ University of Texas Health Science Center at San Antonio, Department of Psychiatry and Behavioral Sciences, San Antonio, Texas; § University of Texas at San Antonio, Department of Psychology, San Antonio, Texas

To the Editor:

We are delighted that the authors of the comment share our enthusiasm for emergency department (ED) expertise in the management of suicidality and appreciate their attention to our scoping review methodology. Their primary concern is the exclusion of standard and generally accepted techniques of screening, joint safety planning, patient education, lethal means counseling, follow-up contacts, and the involvement of friends and family. They rightly point out that the Rosen chapter^1^ and the guidelines from which it is derived^2^ should not be taken to define the clinical “standard of care,” which is why we avoided this terminology in our review. However, it is worth noting that the ICAR2E clinical guidelines published by the American College of Emergency Physicians demonstrate almost total overlap with the interventions described in Rosen.^3^,^4^

We concur with the authors’ argument that the interventions described in these textbooks merit further study, especially given their current evidentiary basis.^3^ However, the purpose of our review was to delineate what is on the horizon for the ED management of suicidality and not provide a comprehensive review of interventions that have already been established. Given the lag in publishing, we took the appearance in authoritative textbooks to indicate that the listed interventions are mainstream and, thus, not pertinent to a review of techniques that have been only recently described. Given the concordance of these interventions with current guidelines,^3^ this method appears to have been effective.

The authors of the comment highlight that critical appraisal of individual sources is optional in the PRISMA-ScR checklist,5 and we acknowledge their perspective on its potential contribution in this topical area. We agree fully with their point that crisis response planning shows promise but that existing evidence for widespread adoption is wanting,^6^ as we clearly noted in our discussion and conclusion sections.

Suicidality is a significant clinical challenge in the ED, and we thank the comment authors for acknowledging our review.
